# Carbon-Coated ZnS-FeS_2_ Heterostructure as an Anode Material for Lithium-Ion Battery Applications

**DOI:** 10.3390/ijms232213945

**Published:** 2022-11-11

**Authors:** Perumal Naveenkumar, Munisamy Maniyazagan, Nayoung Kang, Hyeon-Woo Yang, Woo-Seung Kang, Sun-Jae Kim

**Affiliations:** 1 Metal-Organic Compounds Materials Research Center, Sejong University, 209, Neungdong-ro, Gwangjin-gu, Seoul 05006, Korea; 2 Department of Nanotechnology and Advanced Materials Engineering, Sejong University, 209, Neungdong-ro, Gwangjin-gu, Seoul 05006, Korea; 3 Department of Metallurgical and Materials Engineering, Inha Technical College, Incheon 22212, Korea

**Keywords:** hydrothermal, carbon coating, heterostructure, lithium-ion battery, ZnS-FeS_2_@C

## Abstract

The construction of carbon-coated heterostructures of bimetallic sulfide is an effective technique to improve the electrochemical activity of anode materials in lithium-ion batteries. In this work, the carbon-coated heterostructured ZnS-FeS_2_ is prepared by a two-step hydrothermal method. The crystallinity and nature of carbon-coating are confirmed by the investigation of XRD and Raman spectroscopy techniques. The nanoparticle morphology of ZnS and plate-like morphology of FeS_2_ is established by TEM images. The chemical composition of heterostructure ZnS-FeS_2_@C is discovered by an XPS study. The CV results have disclosed the charge storage mechanism, which depends on the capacitive and diffusion process. The BET surface area (37.95 m^2^g^−1^) and lower R_ct_ value (137 Ω) of ZnS-FeS_2_@C are beneficial to attain higher lithium-ion storage performance. It delivered a discharge capacity of 821 mAh g^−1^ in the 500th continuous cycle @ A g^−1^, with a coulombic efficiency of around 100%, which is higher than the ZnS-FeS_2_ heterostructure (512 mAh g^−1^). The proposed strategy can improve the electrochemical performance and stability of lithium-ion batteries, and can be helpful in finding highly effective anode materials for energy storage devices.

## 1. Introduction

Rechargeable secondary batteries are currently one of the market’s most important energy storage technologies. Particularly, lithium-ion batteries (LIBs) are the most common power sources for portable electronic devices. Most importantly, electrode materials are crucial to the battery technology’s ability to store and convert electrical charge [1]. However, because the most used graphite anode has a low theoretical capacity (372 mAh g^−1^) and poor rate capability, developing a novel anode material with high energy/power density is a hot topic in LIB research [2]. Because of their high capacity, various anode materials such as transition metal oxides [3], metal sulfides [4], and metal/nonmetal have been widely investigated as promising candidate anodes for LIBs to meet the demand for increased energy and power density for LIBs. In recent years, transition metal sulfides (TMS), which have high theoretical capacities and are cheap, have turned out to be one of these alternatives [5]. Because of its high capacity, abundance, and low cost, ZnS is thought to be the most likely candidate to replace graphite as an anode for LIBs. Nanostructured ZnS could be used for energy storage, environmental remediation, photo electrolysis, and catalysis [6]. Poor structural stability and conductivity have limited ZnS in energy storage applications [7,8]. Much research has gone into improving the structural stability and conductivity of ZnS. Carbonaceous materials are the most stable matrices for ZnS impregnation, which improves ZnS conductivity owing to its porous architecture and structural stability. However, some factors continue to obstruct the use of ZnS. Another method for stabilizing the structure of ZnS-based electrode materials is to create a heterostructure for lithium-ion batteries [9].

Most interestingly, pyrite FeS_2_, an abundant mineral with low toxicity in the Earth’s crust, has a theoretical specific capacity of 894 mAh g^−1^ when it undergoes a four-electron electrochemical reaction between 0.01 and 3.0 V (vs. Li^+^/Li). Sadly, a large volume expansion occurs during the conversion of FeS_2_ to Fe^0^ and Li_2_S during the discharge process, resulting in a severe pulverization issue [10,11]. Consequently, a new solid electrolyte interphase (SEI) film would form on the newly generated nanoparticles’ surface, resulting in a high degree of polarization. In addition, the ground nanoparticles may escape from their original conductive networks and even detach from the anode, resulting in rapid capacity degradation. In addition, low electrical conductivity and slow ionic diffusion kinetic render the FeS_2_ anode’s rate capability inferior. So, FeS_2_ anodes need to be changed right away to make them last longer and increase their rate capability [12,13]. Numerous efforts have been made thus far to address the aforementioned issue with the FeS_2_ anode. In general, the strategy of combining two different metal sulfide nanoparticles with a carbon-based matrix often delivers features of good structural stability with poor volume expansion and boosted electrical conductivity. The uniformly coated carbon layer on the heterostructure significantly improved the Li-ion storage performances.

On the other hand, it was recently found that a heterostructure multi-component TMS can improve the electrochemical reactions and store more energy [14,15]. Herein, heterostructure nanoarrays have several benefits, including rich electroactive sites for redox reactions, an increased electrode–electrolyte contact area, a short electrolyte diffusion path, and attractive synergistic effects between the heterointerface [16,17,18]. Additionally, the junction of the heterosystem could generate the built-in electric charge, which definitely enhanced the electrical conductivity and ion transport, which will lead to better lithium-ion storage properties [17,19]. Herein, the carbon-coated ZnS-FeS_2_ heterostructure is a novel building block for the enhanced electrical conductivity and structural flexibility of long-life lithium-ion battery applications. In this work, two-step hydrothermal methods were used to fabricate the carbon-coated ZnS-FeS_2_ heterostructure for lithium-ion battery applications. ZnS-FeS_2_@C has produced an initial discharge capacity of 1481 mAh g^−1^ at a current density of 0.1 A g^−1^. After 500 cycles, it delivered the specific capacity of 821 mAh g^−1^ in the 500th cycle at a current density of 1 A g^−1^. This method involves merely fabricating the carbon-coated ZnS-FeS_2_ heterostructure-based bimetallic sulphides for outstanding Li-ion storage applications.

## 2. Results and Discussion

### 2.1. Scheme Diagram and Structural Analysis

**Figure 1:**
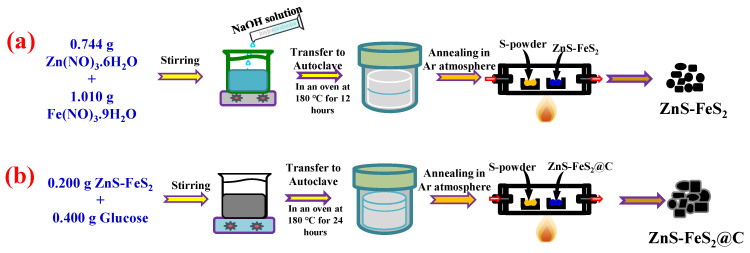
Schematic representation for the (**a**) synthesis of ZnS-FeS_2_ and (**b**) carbon coating of ZnS-FeS_2_ by the hydrothermal method.

The schematic diagram in Figure 1 illustrates the various steps of fabrication of carbon-coated hetero-structured ZnS-FeS_2_@C by a simple hydrothermal method. Firstly, the addition of sodium hydroxide solution to metal ion (Zn^2+^ and Fe^2+^) solution can form the respective metal hydroxides (Zn(OH)_2_ and Fe(OH)_2_). The thiourea react with water and ethanol and release S^2−^ ions. Further, the released S^2−^ ions react with Zn(OH)_2_ and Fe(OH)_2_ to form corresponding ZnS and FeS_2_ during the hydrothermal process. The prepared metal sulphide was carbon coated by glucose using the hydrothermal method, which is displayed in Figure 1b. The carbon-coated heterostructure of ZnS-FeS_2_ was annealed in an argon atmosphere, which improved the crystallinity and completely reduced the unreduced moieties of glucose in the coated-carbon network. This makes the carbon-coated electrode materials more suitable for lithium-ion battery applications. The following reactions take place during the formation of Zn(OH)_2_, Fe(OH)_2_ ZnS, and FeS_2_ [20,21]:

(1) $$Zn^{2+}+2\;{\left(OH\right)}^{-}\;\to \;Zn{\left(OH\right)}_{2}$$

(2) $$Fe^{2+}+2\;{\left(OH\right)}^{-}\;\to \;Fe{\left(OH\right)}_{2}$$

(3) $$Zn{\left(OH\right)}_{2}+Fe{\left(OH\right)}_{2}+3\;S^{2-}\;\to \;ZnS+FeS_{2}+2H_{2}O+O_{2\;\left(↑\right)}$$

**Figure 2:**
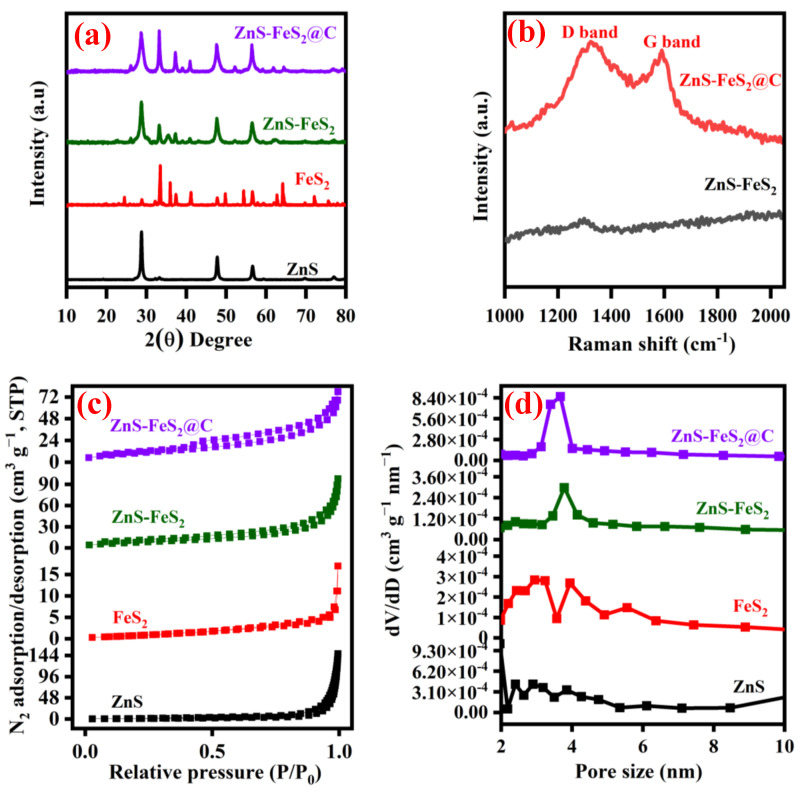
(**a**) XRD pattern; (**b**) Raman spectra of ZnS-FeS_2_ and ZnS-FeS_2_@C; (**c**) N_2_ adsorption/desorption isotherm plot; and (**d**) pore size distributions of ZnS, FeS_2_, ZnS-FeS_2_, and ZnS-FeS_2_@C.

Figure 2a displays the obtained XRD pattern of ZnS, FeS_2_, ZnS-FeS_2_, and ZnS-FeS_2_@C. The obtained XRD patterns have clearly exposed electrode material’s crystallinity and phase purity. The obtained XRD pattern of ZnS has diffraction peaks at 28.9, 33.4, 47.5, 56.6, 69.8, and 77.2°, which are attributed to the (0054), (1037), (1073), (1154), (0273), and (0291) planes of the ZnS rhombohedral crystal system (JCPDS NO: 01-083-1700). As shown in Figure 2a, pyrite FeS_2_ shows diffraction peaks at 28.7, 33.2, 37.3, 40.8, 47.4, 56.2, 59.2, 61.8, 64.3, 76.6, and 78.9°, which correspond to the (111), (200), (210), (211), (220), (311), (222), (023), (321), (331), and (420) planes with the cubic crystal system (42-1340) [10,22]. However, the heterostructure of ZnS-FeS_2_ and ZnS-FeS_2_@C contains the diffraction peaks of both zinc sulphide and iron sulfide. The carbon-coated ZnS-FeS_2_ has strong diffraction peaks, which exposed a crystalline nature. The average crystalline size of the electrode materials was calculated by the Scherrer formula. The sizes of ZnS, FeS_2_, ZnS-FeS_2_, and ZnS-FeS_2_@C were 28.5 nm, 47.92 nm, 25.74 nm, and 28.90 nm, respectively. No more impurity peaks were observed in the electrode materials, revealing the phase purity of the products. Figure 2b shows the Raman spectra of ZnS-FeS_2_ and ZnS-FeS_2_@C. As can be seen, ZnS-FeS_2_@C shows the two peaks at 1322 cm^−1^ and 1590.6 cm^−1^ corresponding to the D band and G bands, respectively. The intensity ratio of the D and G band is 1.03 for carbon coated heterostructure of ZnS-FeS_2_. This suggests that carbon coating exists in the partially graphitic and disordered carbon network. Further, it may be beneficial to the improvement in conductivity of heterostructure ZnS-FeS_2_ nanomaterials [8]. Moreover, the surface area and pore structure are a valuable parameter to determine the electrochemical kinetics of electrode materials. The N_2_ absorption/desorption quantities of carbon-coated ZnS-FeS_2_ heterostructure is higher than ZnS, FeS_2_, and ZnS-FeS_2_, which is shown in Figure 2c. The BET surface area value of ZnS-FeS_2_@C is 37.95 m^2^g^−1^, which is higher than ZnS-FeS_2_ (29.20 m^2^g^−1^), ZnS (4.55 m^2^g^−1^), and FeS_2_ (3.23 m^2^g^−1^). The surface area of heterostructure is significantly enhanced by carbon coating using glucose. Additionally, in Figure 2d, the pore size of ZnS, FeS_2_, ZnS-FeS_2_, and ZnS-FeS_2_@C is 1.43 nm, 1.42 nm, 0.87 nm, and 0.87 nm, respectively. The nano porous nature and high surface area of electrode materials are beneficial for the better electrochemical activities in lithium-ion batteries [8,23].

**Figure 3:**
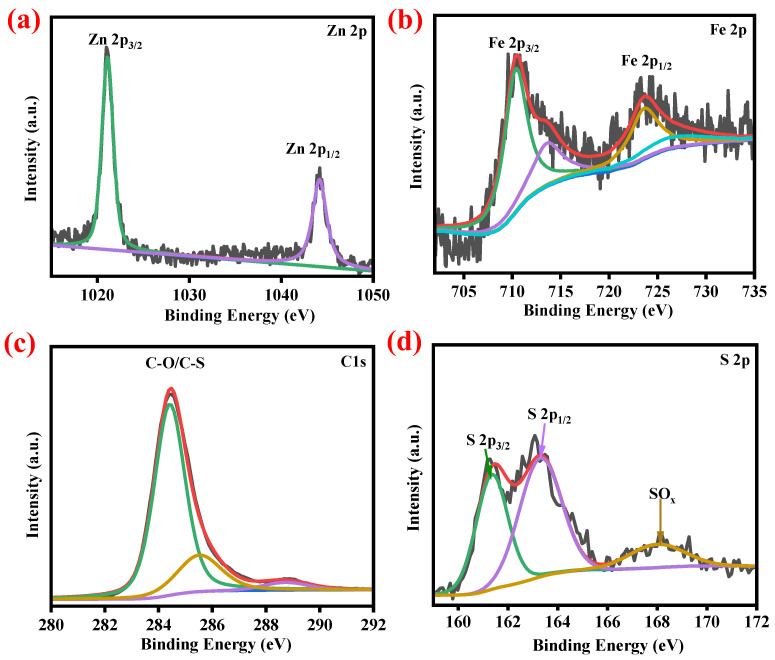
XPS spectra of ZnS-FeS_2_@C: (**a**) Zn2p, (**b**) Fe2p, (**c**) C1s, and (**d**) S2p.

XPS measurements are carried out to understand the elements and their chemical states of electrode materials. As shown in Figure S1, the survey spectrum contained the Zn, Fe, S, and C elements in ZnS-FeS_2_@C. The high-resolution spectrum of Zn 2p (Figure 3a) had two predominant peaks at 1021.04 eV and 1044.2 eV, corresponding to Zn 2p_3/2_ and Zn 2p_1/2_, respectively. This represents the zinc that exists in +2 state in ZnS-FeS_2_@C [24,25,26]. As for the Fe 2p spectrum in Figure 3b, it has two major peaks around 710 eV and 725 eV, corresponding to FeS_2_ satellite peaks. The deconvoluted peaks of Fe 2p spectrum at 710.5 eV, 723.7 eV, and 713.8 eV, 726.5 eV are attributed to the Fe 2p_3/2_ and Fe 2p_1/2_ orbitals, which correspond to Fe^2+/3+^ states [10,12,27]. Figure 2c displays the carbon 1s spectrum, which holds the three deconvolution binding energies at 284.5 eV, 285.4 eV, and 288.8 eV, designated to C-C&C=C, C-O/C-S, and C=O/O-C=O, respectively [10,27,28]. However, the binding energies at 161.3 eV and 163.4 eV were attributed to the S 2p_3/2_ and S 2p_1/2_ orbitals with S^2−^state, respectively. The peak at 168.12 eV in S2p spectrum (Figure 2d) revealed the surface oxidation of electrode materials [28,29]. These results suggest that the existence of a carbon layer in the heterostructure of ZnS-FeS_2_ may be helpful to enhance the structural flexibility with a good conductive carbon network system. It may long establish the electrochemical activities in lithium-ion storage applications.

### 2.2. Morphological Analysis

**Figure 4:**
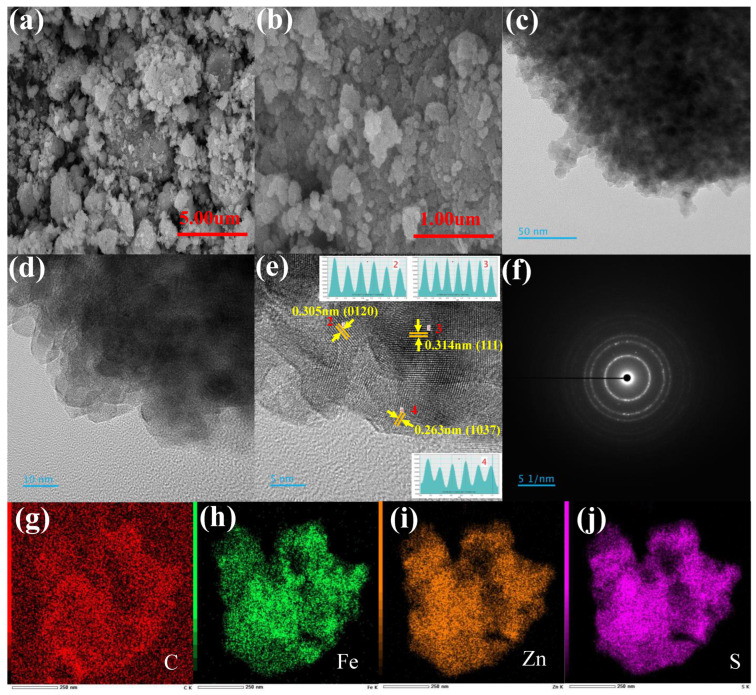
(**a**,**b**) FE-SEM images and HRTEM images with (**c**) low magnification, (**d**,**e**) high magnification, (**f**) SAED pattern, and (**g**–**j**) EDS mapping images of ZnS-FeS_2_@C.

The surface morphology of ZnS, FeS_2_, ZnS-FeS_2_, and ZnS-FeS_2_@C was investigated by SEM, with images displayed in Figure 4, Figures S2 and S3. Figure S2a–c shows the nanoparticle morphology of ZnS powder sample. The EDS mapping exposed the uniform distribution of zinc and sulphur elements in Figure S2d,e. The elemental map sum spectrum reveals the presence of zinc and sulphur elements in Figure S2f. SEM images of FeS_2_ display the stacked plate-like morphology with respective iron and sulphur elements in composition in Figure S3a–f. The heterostructure of ZnS-FeS_2_ depicts the agglomerated nanoparticle morphology in Figure S4a,b. Further clear observation of heterostructure ZnS-FeS_2_ morphology in HRTEM images has demonstrated FeS_2_ in the plate-like structure, but ZnS in the uniform nanoparticle structure in Figure S4c,d. The high-resolution images in Figure S4e at 10 nm contain the two different lattice fringes, which are associated with ZnS and FeS_2_ phases. The SAED pattern ZnS-FeS_2_ has clear diffraction spots, which confirm the crystalline nature of ZnS-FeS_2_. Figure S4g–i images were exposed the consistent distribution of Fe, Zn, and S elements.

Moreover, carbon-coated heterostructure ZnS-FeS_2_ exhibits the same agglomerated nanoparticle morphology for the prepared powder sample in Figure 4a,b. Figure 4c,d shows the HR-TEM images of ZnS-FeS_2_@C, which exposed that the heterostructured ZnS-FeS_2_ nanoparticles are coated with a thin layer carbon by the hydrothermal method. The hydrothermally carbon-coated layer is annealed to improve the crystallinity of the final composition. The high magnification image at 5 nm (Figure 4e) comprise different kinds of lattice fringes, which associated with 0.305 nm and 0.263 nm d spacing, corresponding to (0120) and (1037) planes of ZnS, and 0.314 nm d spacing, corresponding to the (111) plane of FeS_2_ nanoparticles. The selected area electron diffraction image in Figure 4f demonstrates a clear diffraction pattern, which further agrees with the crystalline nature of ZnS-FeS_2_@C. The uniform distribution C, Zn, Fe, and S elements in ZnS-FeS_2_@C is confirmed by elemental mapping images in Figure 4g–j. More specifically, the TEM results of ZnS-FeS_2_ and ZnS-FeS_2_@C revealed an average particle size of 22 nm and 25 nm, respectively. These results are in good agreement with the calculated average grain size of electrode materials obtained from the XRD results. However, this study reveals the presence of carbon-coated heterostructured nanoparticles, which is beneficial for a higher amount of lithium-ion intercalation/de-intercalation rate with improved structural stability.

### 2.3. Electrochemical Analysis

**Figure 5:**
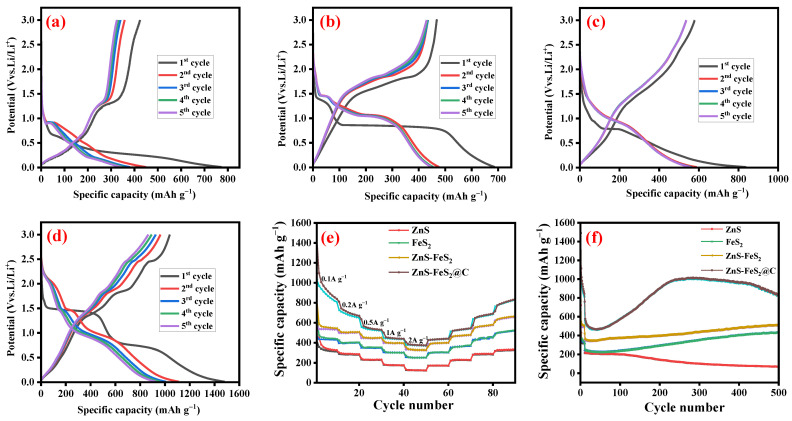
Charge–discharge voltage profiles of (**a**) ZnS, (**b**) FeS_2_, (**c**) ZnS-FeS_2_, and (**d**) ZnS-FeS_2_@C, as well as (**e**) Rrte capability study of all the electrode materials and (**f**) cyclic performance of ZnS, FeS_2_, ZnS-FeS_2_, and ZnS-FeS_2_@C at a current density of 1 A g^−1^ over 500 cycles.

In order to strengthen the structural features of the carbon-coated ZnS-FeS_2_ heterostructure by galvanostatic charge/discharge studies, cyclic voltammetry study and electrochemical impedance spectroscopy were studied. The above-mentioned studies were carried out by assembled 2032 half cells. Figure 5a displays the initial galvanostatic charge/discharge cycles of ZnS nanoparticles. It delivered the first cycle discharge/charge capacity of 773/424 mAh g^−1^ at a current density of 0.1 A g^−1^, with a coulombic efficiency of 55%. The subsequent cycle curve shape was more intact with each other, which increased to a coulombic efficiency of 94% in the fifth cycle, with a specific capacity of 344 mAh g^−1^. Figure 5b displays the discharge/charge curve of FeS_2_, which delivered the specific capacity of 687/469 mAh g^−1^ at a current density of 0.1 A g^−1^. The initial coulombic efficiency of FeS_2_ was 68%; in the continuous cycle, it was increased to reach the maximum (96.3% in the fifth cycle). Figure 5c,d shows the charge/discharge plot of heterostructure ZnS-FeS_2_ and ZnS-FeS_2_@C at a current density of 0.1 A g^−1^. The charge/discharge capacities of the first cycle were 579/839 mAh g^−1^ and 1039/1481 mAh g^−1^ for heterostructure ZnS-FeS_2_ and ZnS-FeS_2_@C, respectively, with the corresponding coulombic efficiency of 69 and 70%, respectively. The capacity loss in the very first cycle corresponds to solid electrolyte interphase film formation [22,30]. The subsequent cycle plot was overlapped, which represents the good lithium-ion insertion/extraction behaviour of ZnS-FeS_2_@C.

The rate capability of ZnS, FeS_2_, ZnS-FeS_2_, and ZnS-FeS_2_@C was measured at various current densities, as shown in Figure 5e. The ten continuous cycles were performed at every current density to estimate the average specific capacity of ZnS-FeS_2_@C, which was 921, 700, 540, 447, and 382 mAh g^−1^ at current densities of 0.1, 0.2, 0.5, 1, and 2 A g^−1^, respectively. Again, the current density was switched to 0.1 A g^−1^, and it delivered the specific capacity of 811 mAh g^−1^. Additionally, the ZnS, FeS_2_, and heterostructure ZnS-FeS_2_ achieved a discharge capacity of 329/129.4 mAh g^−1^, 443/254 mAh g^−1^, and 650/328 mAh g^−1^, respectively, at a current density of 0.1/2 A g^−1^. Moreover, the rate capability plot expressed an improved specific capacity when the current density reaches 0.1 A g^−1^. The reactivation of electrode materials leads to an attained maximum specific capacity.

Further, Figure 5f displays the long-term cycle stability of the electrode materials at a current density of 1 A g^−1^. The ZnS nanoparticle has shown the 1st and 500th cycle discharge capacity of 241 and 68 mAh g^−1^, respectively. This plot conveyed that ZnS has a decreasing trend of discharge capacity, caused by the poor electrical conductivity of ZnS. The pristine plate like FeS_2_ shows a discharge capacity of 429 mAh g^−1^ in the 500th cycle. This plot has revealed the increasing specific capacity of FeS_2_ from the 100th cycle, achieved by a high amount of reactivation of electrode materials. The heterostructured ZnS-FeS_2_ delivered a specific capacity of 512 mAh g^−1^ in the 500th cycle. Additionally, this cyclic stability plot of ZnS-FeS_2_ has revealed the flatly increasing trend compared with pristine FeS_2_. Finally, the carbon-coated heterostructured ZnS-FeS_2_ nanoparticles delivered a specific capacity of 567 mAh g^−1^ in first cycle, which was increased to 821 mAh g^−1^ in 500th continuous cycle, with a coulombic efficiency of around 100%. The cyclic graph of ZnS-FeS_2_@C was depicted the initial cycles it was decreasing capacity which corresponds to the formation of a stable SEI film and activation electrode materials. From the 50 to 250th cycle, the specific capacity was well improved to reach a maximum; after the 250th cycle, it underwent stabilization up to the 435th cycle. After the 435th cycle, it underwent the capacity fading; finally, it reached a specific capacity of 821 mAh g^−1^ in the 500th cycle. The hydrothermal carbon coating of the heterostructure definitely improved the electrical conductivity and prevented the pulverization of electrode materials, leading to good structural flexibility. The reactivation of electrode materials was a commonly noted behaviour in metal-based nanomaterials and carbon-based materials [31,32]. The obtained specific capacity value was compared with reported ZnS- and FeS_2_-based anode materials in LIB applications, which are given in Table 1.

Further, the electrochemical activity of the electrode materials was evaluated in 2032 coin-type half cells. The CV of all of the electrode materials was conducted between 0.01 and 3.0 V versus Li+/Li at various scan rates from 0.2 to 1.2 mV s^−1^. As shown in Figure S5a, at the very first cathodic scan of ZnS, 0.25, 0.55, 0.67, and 1.36 V correspond to the decomposition of ZnS and formation of Li_2_S, LiZn alloy, and SEI film, respectively. The anodic scan of ZnS shows the peak current at 0.76 and 0.35 V, which were associated with the decomposition of Li_2_S, LiZn alloy, and ZnS formation [36,37,38].

(4) $$ZnS+2Li^{+}+2\;e^{-}\;\to \;Zn+Li_{2}S$$

(5) $$Zn+Li^{+}+e^{-}\;\to \;LiZn$$

The above redox reactions occurred in the continuous scans, corresponding to the multistep alloying/dealloying process of lithium-zinc alloys [39]. In Figure S6a, the CV curve of plate-like FeS_2_ is displayed, and the cathodic scan of the FeS_2_ contained peak current at 1.95 V corresponds to the decomposition of FeS_2_ and formation of Li_2_S and Fe. The oxidation peak current at 0.91 and 1.34 V corresponds to conversion of Fe to FeS_2_. The electrochemical reactions are described below in Equations (4) and (5) [12,22,27].

(6) $$FeS_{2}+2Li^{+}+2\;e^{-}\;\to \;Zn+Li_{2}FeS_{2}$$

(7) $$Li_{2}FeS_{2}+2Li^{+}+2\;e^{-}\;\to \;Fe+Li_{2}S\;$$

**Figure 6:**
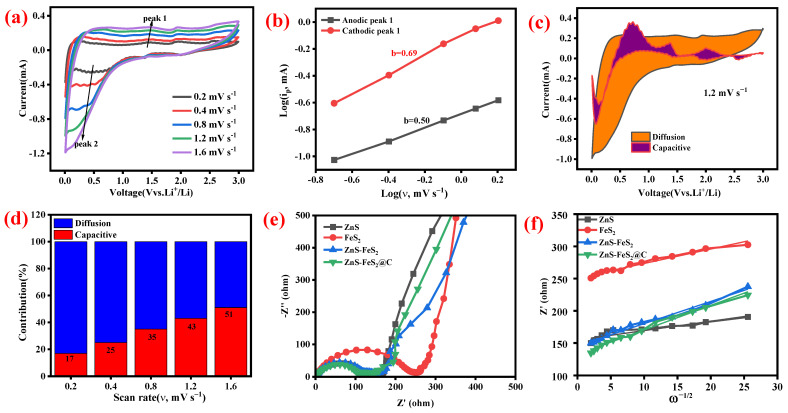
(**a**) CV curves of the ZnS-FeS_2_@C electrode at different scan rates from 0.2 to 1.6 mV s^−1^. (**b**) Linear fitting of log (peak current) versus log (scan rate) plot of ZnS-FeS_2_@C. (**c**) Capacitive and diffusion contribution of ZnS-FeS_2_@C electrode @ 1.2 mV s^−1^. (**d**) Percentage of capacitive and diffusion contribution ratio of ZnS-FeS_2_@C electrode at different rates. (**e**) Nyquist plot of ZnS, FeS_2_, ZnS-FeS_2_, and ZnS-FeS_2_@C. (**f**) Straight line fitting of Z’ versus ω^−1/2^.

Furthermore, the heterostructured ZnS-FeS_2_ in Figure S7a has a strong reduction/oxidation peak at 1.58/0.82 V, corresponding to the decomposition of ZnS and FeS_2_ as well as formation of Li_2_S and their reverse reactions. The carbon-coated ZnS-FeS_2_ has smothered redox peaks in Figure 6a, and the subsequent cycles at different scan rates also have an identical shape. This represents the good reversibility of ZnS-FeS_2_@C electrode material. Additionally, the dependence of charge storage mechanism was evaluated by power law [40]:

(8) $$i=av^{b}$$

where *i* is the peak current, *ν* is the scan rate (mV s^−1^), and a and b are constant parameters. A b value close 1 indicates that the capacitive behaviour is dominant, while *a b* value close to 0.5 represents that the charge storage depends on the diffusion process. Figure S5b displays that the *b* valves of anodic peak 1 to 3 and cathodic peak 1 to 2 are 0.63, 0.66, 0.83, 0.68, and 0.79, respectively, representing that the charge storage mechanism majorly depends on the combined process. The *b* value of FeS_2_ in Figure S6b indicates that the charge storage mechanism corresponds to a diffusion-controlled process. The heterostructured ZnS-FeS_2_ has a b value of 0.72/0.87, attributed to the combined charge/discharge mechanism in Figure S7b. Here, Figure 6b displays that the b value of the anodic and cathodic peak is 0.5/0.69, which characteristics the combined charge storage mechanism. In addition, the exact contribution of the current response was predicted by the following equation:

(9) $$i=k_{1}v+k_{2}v^{0.5}$$

where *k*_1_ and *k*_2_ are parameters and *k*_1_*v* and *k*_2_*ν*^0.5^ are assigned to capacitive and diffusion-controlled contribution, respectively. Figures S5c,d, S6c,d and S7c,d display the charge contributions of ZnS, FeS_2_, and ZnS-FeS_2_ at various scan rates, respectively. When increasing the scan rate from 0.2 to 1.6 V, the capacitive contributions also increase. Figure 6c reveals that the carbon-coated heterostructured ZnS-FeS_2_ have 57% of the diffusion process in the total capacity at a scan rate of 1.2 mV s^−1^. Figure 6d reveals that the capacitive behaviour increased from 17 to 51%, with a corresponding scan rate of 0.2 to 1.6 mV s^−1^. The combined charge storage mechanism of ZnS-FeS_2_@C composition has revealed excellent cycle stability, which agrees with the good cycle stability nature of the carbon-coated ZnS-FeS_2_ heterostructure.

To further investigate the electrochemical performance of the electrode materials at room temperature, an EIS study was conducted. As depicted in Figure 6f, the Nyquist plot was comprised of a semicircle in the high frequency region attributed to the charge transfer resistance (R_ct_), where the y-axis intercept in the x-axis represents the solution resistance (R_s_) and tilted line at a low frequency corresponds to the Warburg impedance (σ). The fitted equivalent circuit of the Nyquist plot is shown in Figure S8 and the fitted parameters are described below. The solution resistance of the carbon-coated heterostructure is 1.12 Ω, which lower than heterostructured ZnS-FeS_2_ (1.2 Ω). The charge transfer resistance of ZnS-FeS_2_@C is 137 Ω, which is lower than that of other electrode materials. The lower charge transfer resistance of ZnS-FeS_2_@C is beneficial to the high amount of lithium-ion or electron transport between the anode and cathode. It leads to an excellent performance in lithium-ion battery applications. As shown in Figure 6f, the Warburg factor (σ) value of ZnS, FeS_2_, ZnS-FeS_2_, and ZnS-FeS_2_@C is 154.7, 251, 145.6, and 132.3, respectively. A lower Warburg factor value indicates better ionic conductivity of ZnS-FeS_2_@C. The lower R_s_, R_ct_, and σ values of ZnS-FeS_2_@C have endorsed the obtained good rate capability and cyclic stability behaviour [41].

On the basis of the preceding analysis, the significantly enhanced electrochemical performance is primarily attributable to the amplified conductivity, enhanced e^−^/Li^+^ transfer efficiency, and retarded volume expansion during the electrochemical process, which is closely related to the structural advantages of the carbon-coated heterostructured ZnS-FeS_2_ and can be assumed to be due to the following aspects: (1) the presence of nano-structured materials can shorten the ion diffusion path length, (2) the synergistic effect between the heterostructure interface, (3) the carbon coating can improve the electrical conductivity and structural stability of ZnS-FeS_2_, (4) the combined charge storage mechanism is helpful for long cyclic performance, and (5) the lower charge transfer and ionic diffusion resistance may lead to an excellent rate capability and improve the capacity of the electrode materials. The as-prepared carbon-coated ZnS-FeS_2_ heterostructure anode materials exhibit excellent electrochemical performance in higher lithium-ion storage applications owing to the above-mentioned factors.

## 3. Methods and Materials

### 3.1. Chemicals

Zinc nitrate hexahydrate (Zn(NO)_3_ 6H_2_O), iron nitrate nonahydrate (Fe(NO)_3_ 9H_2_O), sodium hydroxide (NaOH), thiourea ((NH_2_)_2_CS), ethanol (C_2_H_5_OH), sulphur powder (S) was purchase from Sigma Aldrich (Seoul, Korea) and Millipore water was used in this work. The chemical used these experiments without further purification.

### 3.2. Synthesis of ZnS, FeS_2_, and ZnS-FeS_2_ Heterostructure

In a typical synthesis of ZnS-FeS_2_, 0.744 g Zn(NO)_3_ 6H_2_O and 1.01 g of Fe(NO)_3_ 9H_2_O were dissolved in 40 mL of ethanol. Then, 2.0 g of NaOH in 40 mL water was added to the above metal ion solution dropwise. After the complete addition of NaOH, it was continuously stirred for an hour. To the above mixture, 0.762 g of thiourea was added and continuously stirred for an hour. After that, it was transferred into a 120 mL Teflon-lined stainless-steel autoclave. It was kept in an oven at 180 °C for 12 h. The final products were centrifuged and washed with water and ethanol to remove impurities. The hydrothermally prepared ZnS-FeS_2_ was annealed at 600 °C for 2 h in the Ar atmosphere. The bare ZnS and FeS_2_ were synthesized using the same procedure using corresponding precursors of 1.487 g Zn(NO)_3_ 6H_2_O and 2.02 g of Fe(NO)_3_ 9H_2_O, respectively.

### 3.3. Carbon Coating of ZnS-FeS_2_

Here, 200 mg of ZnS-FeS_2_ was dispersed in 40 mL water. Then, 400 mg of glucose in 40 mL water was added dropwise and continuously stirred for an hour. It was transferred to 120 mL of Teflon-lined stainless-steel autoclave and kept in an oven at 180 °C for 24 h. The final product of carbon-coated ZnS-FeS_2_ was collected by centrifuge process, and it was washed with ethanol and water several times. It was annealed at 600 °C for 2 h in an Ar atmosphere. Further, it was used for the analysis.

### 3.4. Material Characterizations

The crystal structure and phase purity of ZnS, FeS_2_, ZnS-FeS_2_, and ZnS-FeS_2_@C were investigated by X-ray diffraction analysis using PANalytical instrument with Cu Kα radiation. Raman spectra of ZnS-FeS_2_ and ZnS-FeS_2_@C were recorded using Renishaw Inc. (Wotton-under-Edge, UK), Raman instrument. N_2_ adsorption/desorption isotherm spectrum was recorded using nano POROSITY-HQ Mirae Instruments (Seoul, Korea). The chemical state of elements in the final composition was examined by X-ray photoelectron spectroscopy equipped with Al Kα radiation (Thermo Scientific Inc., Waltham, MA, USA). The surface morphology of the materials is noted by FE-SEM (SU08010) Tokyo, Japan and HR-TEM system equipped with an energy-dispersive X-ray spectroscopy system operated at an accelerating voltage of 200 kV (JEM-ARM200F). Electrodes are made up using a 70:10:20 ratio of active material, sodium carboxy methyl cellulose, and super P with 1 mL of water as solvent. The slurry was coated on the surface of copper foil, which is dried in a vacuum oven overnight. The Li-ion storage capability was investigated by an assembled 2032-type coin cell. It was fabricated in an Ar-filled glove box using Li metal and polypropylene as counter/reference electrodes and a separator with 1.2 M LiPF_6_ in ethylene carbonate and dimethyl carbonate with 3 wt% of vinyl carbonate as an electrolyte additive. Cyclic voltammetry was carried out with a potential window of 0.01 to 3.0 V using different scan rates 0.2 to 1.2 mVs^−1^. Electrochemical impedance spectroscopy was analysed using the frequency range from 0.1 kHz to 100 mHz with an applied amplitude of 5 mVs^−1^ in biologic instrument. Cycle stability and rate capability of (lithium-ion storage and conversion) were measured using a WonAtech battery tester (WBCS3000S WonAtech Co., Seoul, Korea).

## 4. Conclusions

In conclusion, a facile composition of the ZnS-FeS_2_@C heterostructure is prepared by the hydrothermal method for high-performance LIB anode materials. The crystalline carbon-coated ZnS-FeS_2_ heterostructure has a high surface area of 37.95 m^2^g^−1^, which is higher than that of the heterostructured ZnS-FeS_2_. The nanoparticle morphology of ZnS and the plate-like morphology of FeS_2_ have their own advantages of higher hetero interface as well as shorter ion diffusion paths. During the charge–discharge process, ZnS-FeS_2_@C has followed both capacitive and diffusion mechanisms, which leads to good rate capability behaviour. These features have definitely improved the Li-ion storage-ability, in terms of the ZnS-FeS_2_@C, which delivered an initial discharge capacity of 1481 mAh g^−1^ at a current density of 0.1 A g^−1^. Additionally, ZnS-FeS_2_@C nanoparticles delivered a specific capacity of 567 mAh g-1 in the first cycle, which is increased to 821 mAh g^−1^ in the 500th cycle at a current density of 1 A g^−1^. Overall, this work offers more insights that novel heterostructures will have broad application prospects in lithium-ion storage applications.

## Figures and Tables

**Table 1:** Comparison of Li-ion battery performance of ZnS-FeS_2_@C with other reports.

Materials	Reversible Capacity—(mAh g^−1^)	Current Density—(A g^−1^)	Cycles	Reference
ZnS-FeS_2_@C	821	1	500	This work
FeS_2_/SG	400.1	1	400	[11]
rGO@FeS_2_@C	820.7	1	300	[12]
FeS_2_@NSC/SG	392	2.5	400	[22]
Ni doped Co_9_S_8_@ZnS	758	1	500	[26]
FeS_2_@N/S-C	528	1	1000	[27]
ZnS@NSC-800	571.4	1	1000	[28]
CoxZn1-xS/Co_9_S_8_@ rGO	786	1	1000	[29]
ZnO/ZnS@N-C/CNT	386.6	1	400	[33]
Co_9_S_8_/ZnS@NC	411.2	1	300	[34]
FeS_2_@CNT	750	1	200	[35]

## Data Availability

Not applicable.

## Supplementary Materials

The supporting information can be downloaded at: https://www.mdpi.com/article/10.3390/ijms232213945/s1.
