# Supplementary material for: Carbon-Coated ZnS-FeS2 Heterostructure as an Anode Material for Lithium-Ion Battery Applications
Source: Int J Mol Sci. 2022 Nov 11;23(22):13945. doi: 10.3390/ijms232213945 (PMC9695666; doi:10.3390/ijms232213945)
Supplement: Supplementary file 1 [file ijms-23-13945-s001.zip › ijms-1985379-supplementary.pdf]

Carbon-coated ZnS-FeS_2 hetero-structure as anode material for Lithium-ion battery applications

Perumal Naveenkumar [1], Munisamy Maniyazagan [2], Nayoung Kang [2], Hyeon-Woo Yang [1,2], Woo-Seung Kang 3 and Sun-Jae Kim [1,2,*]

[1] *Metal-Organic Compounds Materials Research Center, Sejong University, 209, Neungdong-ro, Gwangjin-gu, Seoul 05006, Korea*
[2] *Department of Nanotechnology and Advanced Materials Engineering, Sejong University, 209, Neungdong-ro, Gwangjin-gu, Seoul 05006, Korea*
[3] *Department of Metallurgical and Materials Engineering, Inha Technical College, Incheon 22212, Korea*
* *Correspondence: sjkim1@sejong.ac.kr; Tel.: +82-2-3408-3780*

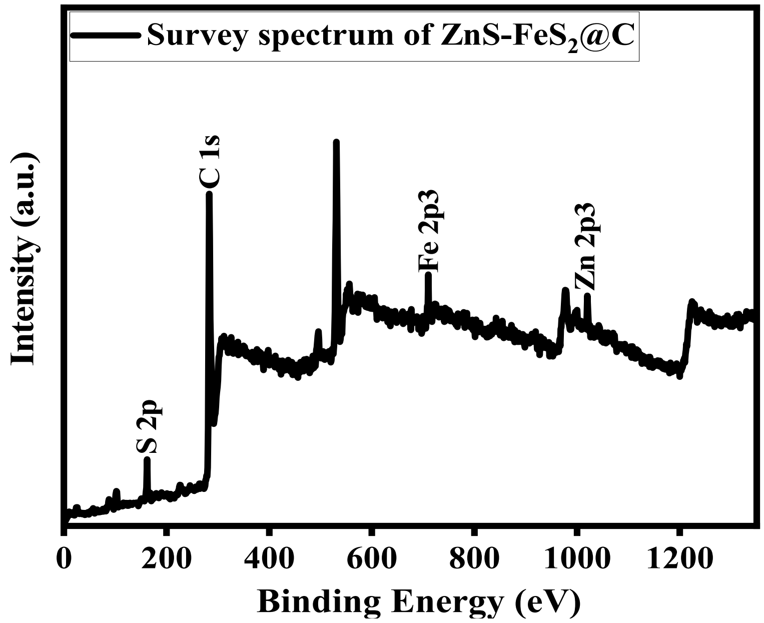

Figure S1. XPS survey spectrum of ZnS-FeS_2@C.

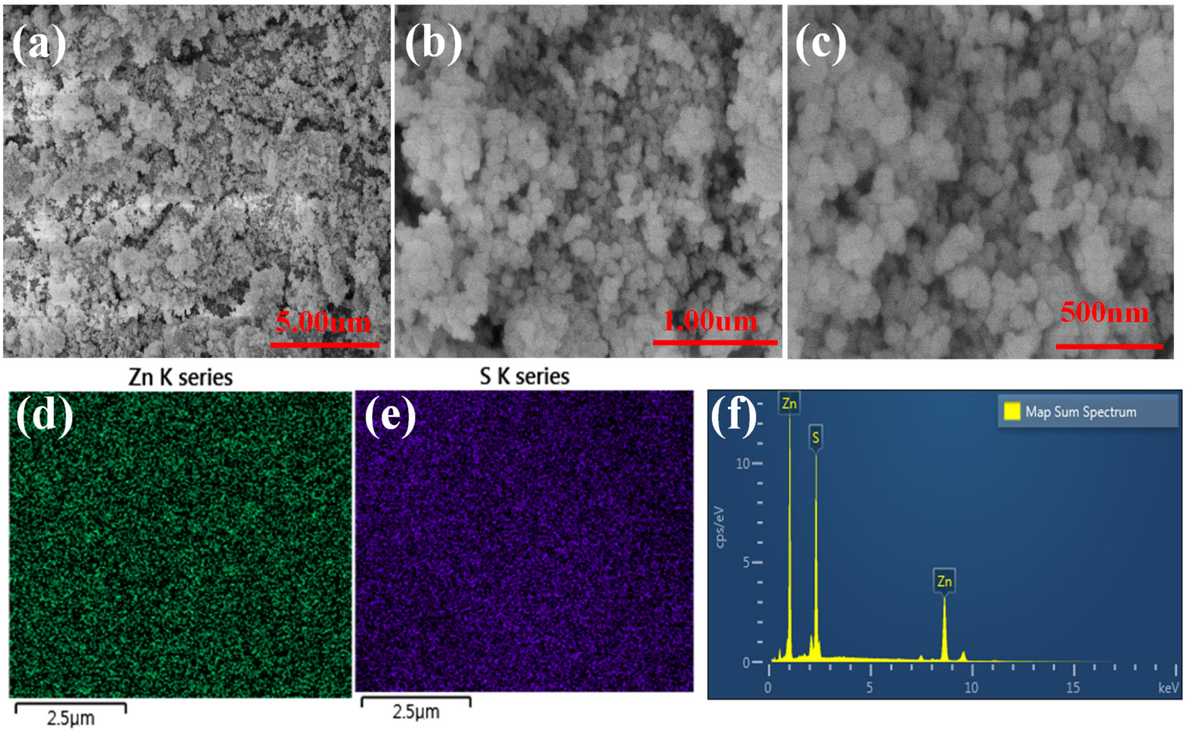

Figure S2. (a-c) FE-SEM images, (d-e) elemental mapping, and (f) elemental mapping spectrum of ZnS.

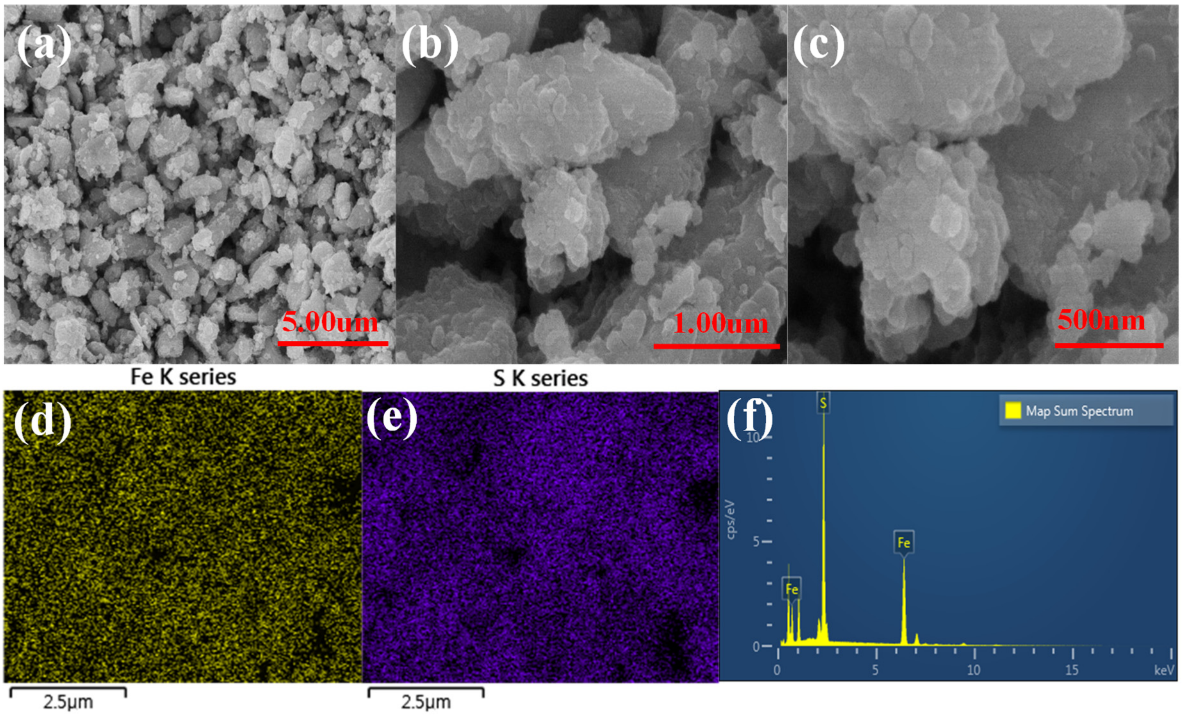

Figure S3. (a-c) FE-SEM images, (d-e) elemental mapping, and (f) elemental mapping spectrum of FeS_2.

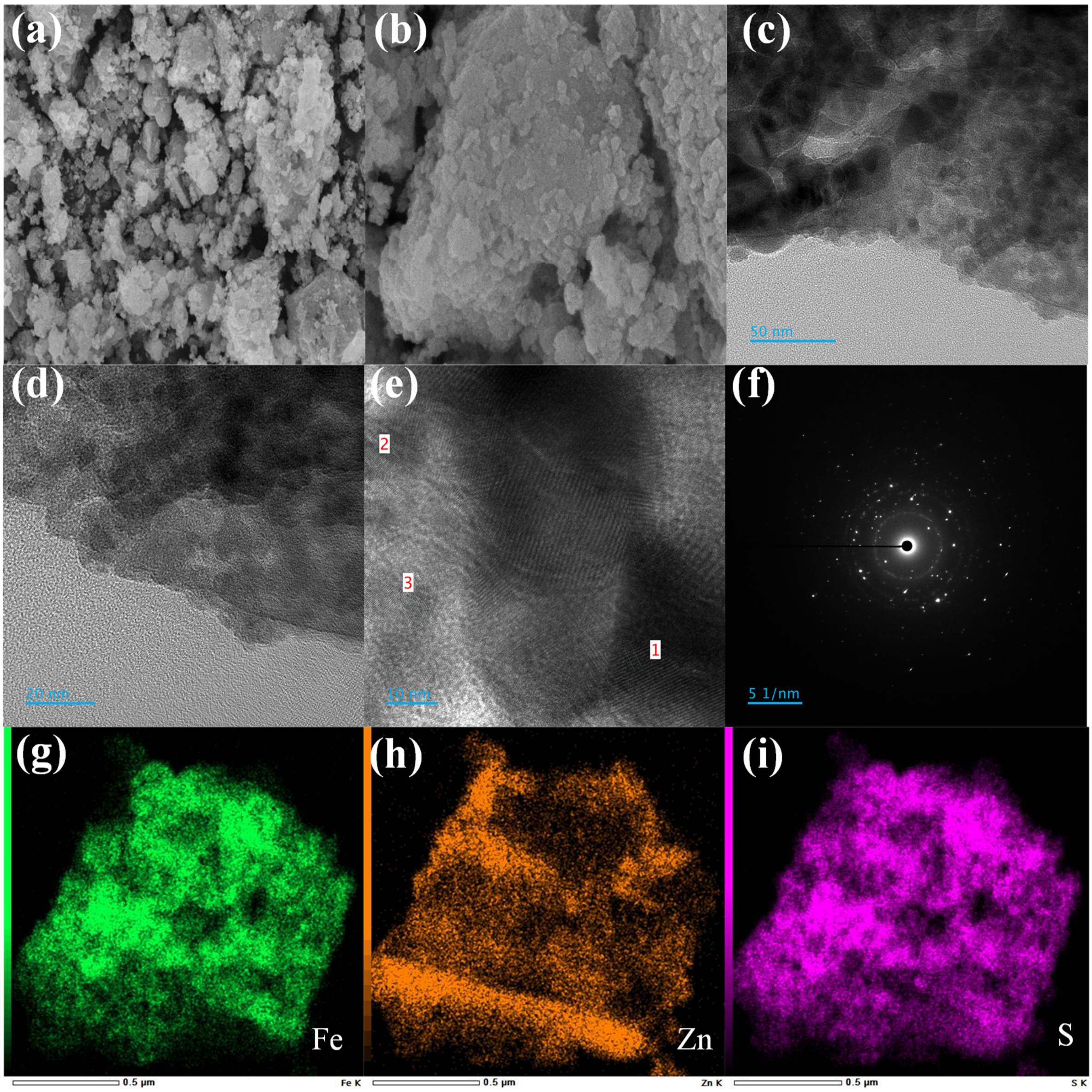

Figure S4. (a-b) FE-SEM images and HRTEM images with (c) Low magnification, (d-e) High magnification, (f) SAED pattern, and (g-j) EDS mapping images of hetero-structured ZnS-FeS_2.

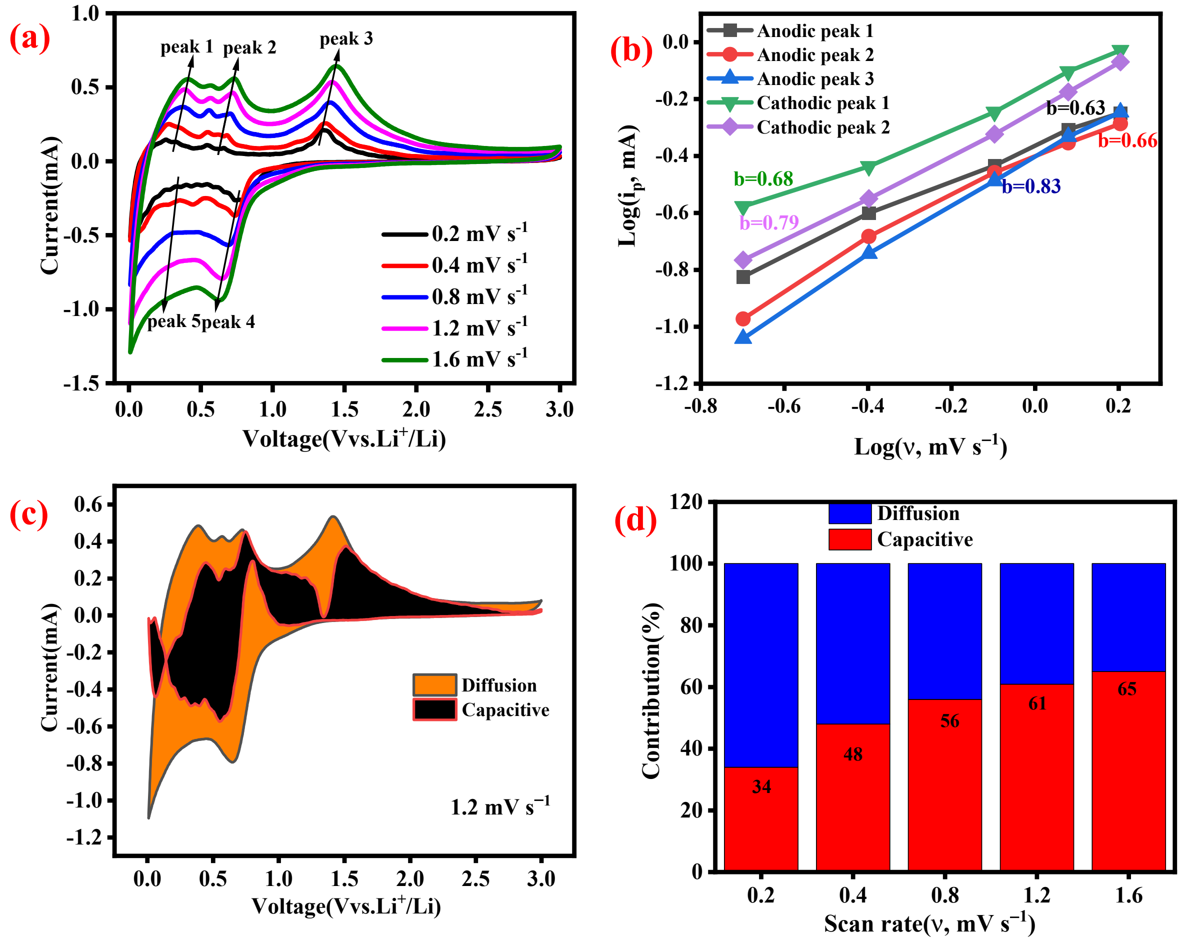

Figure S5. (a) CV curves of the ZnS electrode at different scan rates from 0.2 to 1.6 mV s^{-1}, (b) Linear plot of log peak current versus log scan rate of ZnS, (c) Capacitive and diffusion contribution of ZnS electrode @ 1.2 mV s^{-1}, (d) Percentage of capacitive and diffusion contribution ratio of ZnS electrode at different rates.

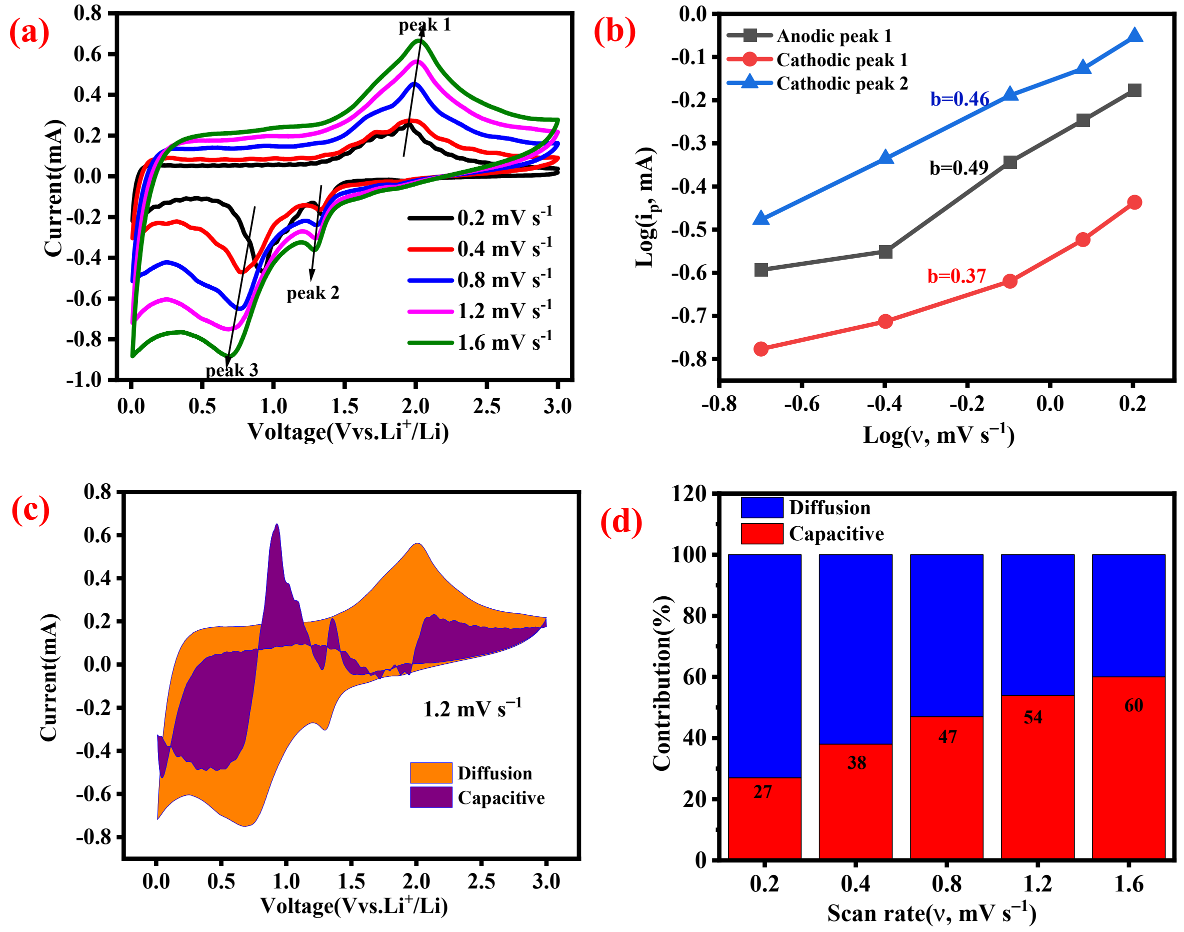

Figure S6. (a) CV curves of the FeS_2 electrode at different scan rates from 0.2 to 1.6mV/s, (b) Linear plot of log peak current versus log scan rate of FeS_2, (c) Capacitive and diffusion contribution of FeS_2 electrode @ 1.2 mV s^{-1}, (d) Percentage of capacitive and diffusion contribution ratio of FeS_2 electrode at different rates.

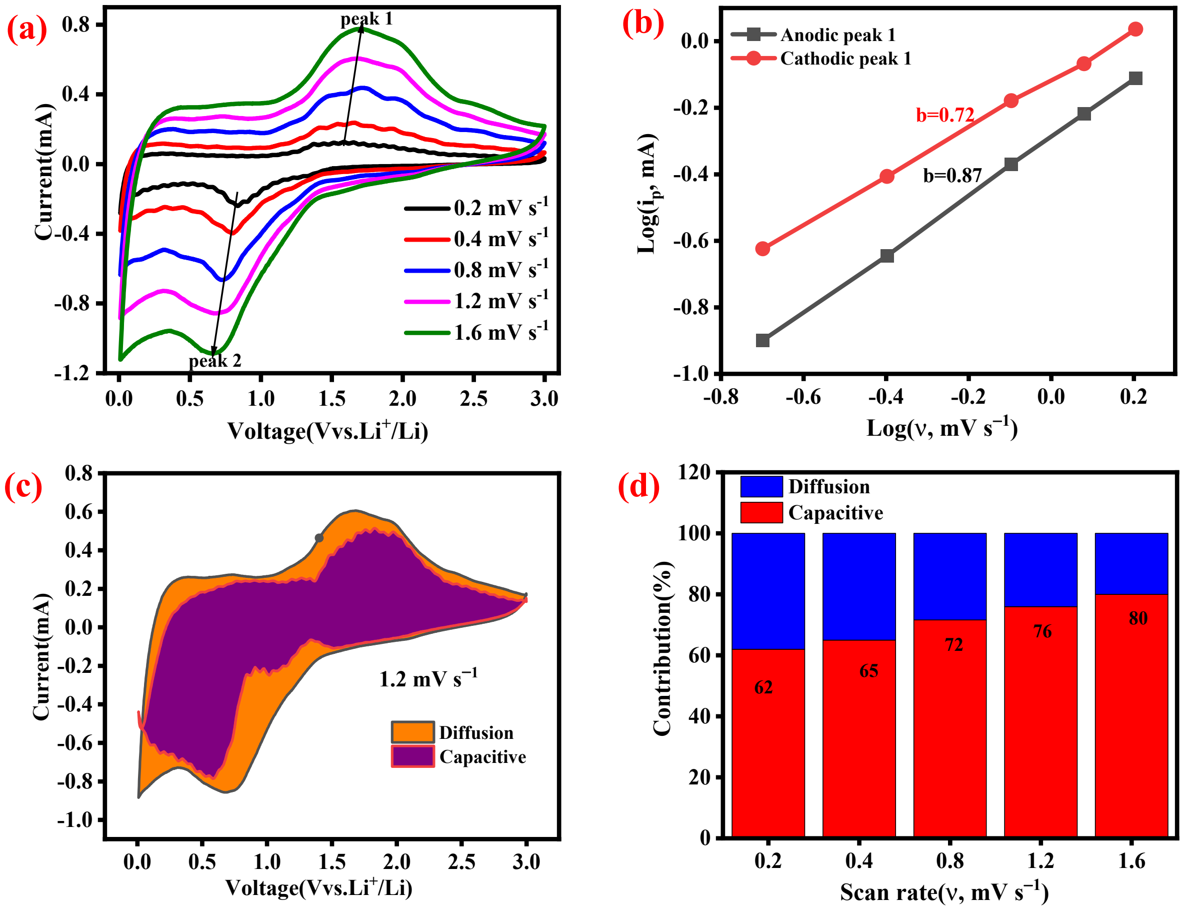

Figure S7. (a) CV curves of the ZnS-FeS$_2$ electrode at different scan rates from 0.2 to 1.6 mV s^{-1}, (b) Linear plot of log peak current versus log scan rate of ZnS-FeS$_2$, (c) Capacitive and diffusion contribution of ZnS-FeS$_2$ electrode @ 1.2 mV s^{-1}, (d) Percentage of capacitive and diffusion contribution ratio of ZnS-FeS$_2$ electrode at different rates.

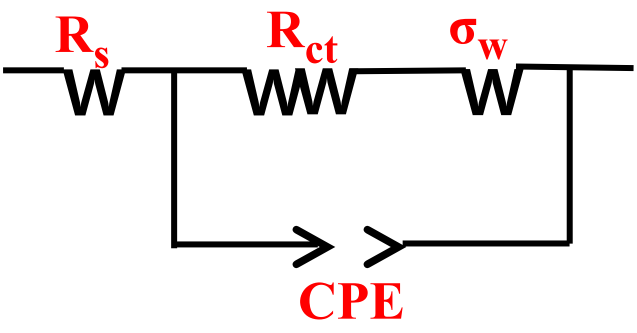

Figure S8. The fitted equivalent circuit of Nyquist plot of all the electrode materials.
